# Cannabinoid Hyperemesis Syndrome Treated With Fosaprepitant: A Case Report

**DOI:** 10.7759/cureus.90477

**Published:** 2025-08-19

**Authors:** Markus D Moore, Saeed K Alzghari, Andrew N Soliman

**Affiliations:** 1 Pharmacy, Texas Health Harris Methodist Hospital Fort Worth, Fort Worth, USA; 2 Pharmacy, Texas Health Hospital Mansfield, Mansfield, USA

**Keywords:** antiemetics, aprepitant, cannabinoid hyperemesis syndrome, cannabinoids, nausea, vomiting

## Abstract

Cannabinoid hyperemesis syndrome (CHS) is a condition in which users of marijuana experience symptoms of nausea and vomiting. Due to the nature of the condition, patients may ultimately be admitted to the hospital for complications such as weakness and fluid loss. The usual treatment options for nausea and vomiting include fluids and a combination of metoclopramide, ondansetron, promethazine, or prochlorperazine. We present a case where fosaprepitant was utilized for intractable nausea and vomiting when other treatment modalities failed for CHS.

## Introduction

Cannabinoid hyperemesis syndrome (CHS) is estimated to affect approximately 2.75 million Americans annually [1]. Reports indicate that emergency department visits related to CHS have doubled in the United States and Canada from 2017 to 2021 and were most prevalent among males aged 16-34 years old [2]. While abdominal pain and vomiting convey a vast variety of differential diagnoses, CHS is a condition described as cyclical nausea, vomiting, and abdominal pain that occurs after ingesting cannabis [3]. This condition is characterized by several years of cannabis use that predates the onset of illness, hyperemesis in a cyclical pattern every few weeks to months, and symptom resolution upon cessation of cannabis ingestion. 

Cannabis elicits its effects at various receptors along the neurologic and endocrinologic pathways [3]. Cannabinoid (CB) receptors are widespread in the brain and are present in the hypothalamus, hippocampus, cerebellum, and vagus nerve. They influence the hypothalamus-pituitary-adrenal axis, which leads to cannabis’s effects on a wide variety of bodily processes, including the vomiting reflex. The gastrointestinal tract has several receptors, including CBs and histamines, among others, which serve to trigger vomiting when stimulated. 

Frequent vomiting due to CHS has multiple complications, ranging from erosion of tooth enamel, dehydration, acute kidney injury, and electrolyte derangement that may require emergency department visits and hospitalization [2]. Aprepitant, an oral neurokinin-1 (NK-1) inhibitor, and fosapreptiant, an intravenous counterpart of aprepitant, have been evaluated in chemotherapy-induced nausea and vomiting, but limited data are available for their use in CHS. Herein, we describe the role of fosaprepitant in the treatment of CHS. 

## Case presentation

A 38-year-old female presented to the emergency room with complaints of nausea and vomiting. She had a history of type 2 diabetes mellitus (T2DM), hypertension, gastroparesis, and seizures. Before her presentation to the emergency department, paramedics found her on the bathroom floor. She was complaining of nausea and vomiting and was given 25 mg of intravenous (IV) promethazine with minor improvement. Her skin was described as cool and clammy, and she denied any trauma, drug use, or alcohol use. This patient also reported that these episodes of nausea and vomiting occurred within the last one to two months. 

On her initial emergency room admission, her initial laboratory findings were unremarkable except for a positive urine drug screen (UDS) for tetrahydrocannabinol (THC). The patient explained to the provider that she was on an unspecified dose of injectable semaglutide and was recently discharged with gastroparesis. Intravenous fluids were administered, and the provider explained that the combination of marijuana use, semaglutide, and her T2DM had likely contributed to her cyclic vomiting episodes. Ultimately, she improved and was discharged.

The patient returned to another nearby emergency room with the same complaints from the day prior and presented with the same symptoms. The patient had a temperature of 99.1 °F and was both hypertensive and tachycardic. The patient's laboratory values were within normal limits except for an elevated white blood cell count and an elevated anion gap (Table 1). Pregnancy testing was negative. Her social history showed that she never used tobacco or experienced second-hand exposure. Similarly, no history of alcohol or drug use was reported by the patient. The patient reported no black or bloody vomitus, but rather it was a clear yellow color. The patient stated that gastroparesis was the cause of the vomiting and initially denied any illicit drugs; however, she did admit to ingesting marijuana gummies roughly three to four days a week and had a positive UDS for CBs. She was treated with 25 mg intravenous (IV) diphenhydramine, 4 mg IV ondansetron, and 10 mg IV prochlorperazine. Afterwards, a 1,000 mL bolus of normal saline and 0.625 mg IV droperidol were given. The patient did not experience relief and continued to vomit during her visit. Subsequently, a dose of 150 mg IV fosaprepitant was ordered and administered. Ultimately, the patient had no more symptoms of nausea and vomiting and was discharged with metoclopramide, pantoprazole, and scopolamine.

**Table 1:** Pertinent laboratory values.

Laboratory test (reference values)	Result
White blood cells (4.0-11.0 k/µL)	18.1
Hemoglobin (11.0-15.0 g/dL)	13.8
Platelets (140-416 k/µL)	198
Sodium (136-145 mmol/L)	138
Potassium (3.5-5.0 mmol/L)	3.8
Bicarbonate (22-31 mmol/L)	21
Anion gap (5-12 mmol/L)	16
Aspartate aminotransferase (15-48 U/L)	20
Alanine aminotransferase (8-56 U/L)	12
Lipase (8-78 U/L)	19

## Discussion

We present a case of CB-induced vomiting with other factors that could have exacerbated the patient’s primary condition. To understand how fosaprepitant works mechanistically, knowing and understanding the physiology behind vomiting is critical. 

CB receptors are found in both the central and peripheral nervous systems. CB_1_ receptors are found in the central nervous system and are predominantly located in the cerebral cortex and cerebellum, whereas CB_2_ receptors are found in the lymphocytes [4]. There are various proposed mechanisms of how cannabis use leads to emesis, but the likely mechanism includes the buildup of lipophilic THC in the brain. THC binds to both CB_1_ and CB_2_ receptors; however, CB_1_ receptors increase dopaminergic activity as well as inhibit gamma-aminobutyric acid in the neurons, which in turn increases the dopaminergic system. These interactions influence the neurons in the midbrain, and one such area includes the ventral tegmental area, which helps suppress the vomiting response. As a result, long-term use of cannabis will downregulate CB_1_ receptors by indirectly increasing dopaminergic activity [4]. These pathways ultimately activate many neurotransmitters. 

Various neurotransmitters are involved in the central nervous system that contribute to the vomiting sensation; the receptors include histamine-1, dopamine-2, serotonin (specifically 5-HT_3_), acetylcholine (muscarinic), and neurokinin (substance P) [5]. To combat the sensation of nausea and vomiting, various receptor antagonists are utilized. Fosaprepitant specifically targets NK-1 receptors, which helps prevent or delay vomiting [6]. NK receptors have a role in chemotherapy-induced vomiting, and fosaprepitant is one such therapy in combination with other medications to treat such conditions [7]. By blocking substance P from activating NK-1 receptors entirely, fosaprepitant interrupts the emetic signaling cascade; hence, this medication is one possible option to use in this indication. 

A previous case report showcased a patient with similar symptoms [8]. This particular patient was a long-term user of marijuana and reported to the emergency department with worsening abdominal pain. The patient was then treated with antiemetics similar to the one’s given to our patient, and when those options failed, they progressed to aprepitant as a final option. Once the medication was given, the patient responded well to the treatment and was promptly discharged. Both of these cases describe how NK-1 inhibitors can be an option to help alleviate CHS, which provides a possible treatment pathway if typical modalities fail.

## Conclusions

In summary, fosaprepitant can be considered for CHS treatment. Providers should understand that CHS manifests into possible emetogenic symptoms that can be treated through various medications. Although fosaprepitant is typically given for chemotherapy-induced nausea and vomiting, this medication seems to be effective as a last resort option in those with CHS.
